# Multidetector Computed Tomography-Based Microstructural Analysis Reveals Reduced Bone Mineral Content and Trabecular Bone Changes in the Lumbar Spine after Transarterial Chemoembolization Therapy for Hepatocellular Carcinoma

**DOI:** 10.1371/journal.pone.0110106

**Published:** 2014-10-16

**Authors:** Miyuki Takasu, Takuji Yamagami, Yuko Nakamura, Daisuke Komoto, Yoko Kaichi, Chihiro Tani, Shuji Date, Masao Kiguchi, Kazuo Awai

**Affiliations:** Department of Diagnostic Radiology, Graduate School of Biomedical Sciences, Hiroshima University, Hiroshima, Japan; Illinois Institute of Technology, United States of America

## Abstract

**Purpose:**

It is well recognized that therapeutic irradiation can result in bone damage. However, long-term bone toxicity associated with computed tomography (CT) performed during interventional angiography has received little attention. The purpose of this study was to determine the prevalence of osteoporosis and trabecular microstructural changes in patients after transarterial chemoembolization (TACE) for hepatocellular carcinoma therapy using an interventional-CT system.

**Materials and Methods:**

Spinal microarchitecture was examined by 64-detector CT in 81 patients who underwent TACE, 35 patients with chronic hepatitis, and 79 controls. For each patient, the volumetric CT dose index (CTDIv) during TACE (CTDIv (TACE)), the dose-length product (DLP) during TACE (DLP (TACE)), and CTDIv and DLP of routine dynamic CT scans (CTDIv (CT) and DLP (CT), respectively), were calculated as the sum since 2008. Using a three dimensional (3D) image analysis system, the tissue bone mineral density (tBMD) and trabecular parameters of the 12th thoracic vertebra were calculated. Using tBMD at a reported cutoff value of 68 mg/cm^3^, the prevalence of osteoporosis was assessed.

**Results:**

The prevalence of osteoporosis was significantly greater in the TACE vs. the control group (39.6% vs. 18.2% for males, *P*<0.05 and 60.6% vs. 34.8% for females, *P*<0.01). Multivariate regression analysis demonstrated that sex, age, and CTDIv (CT) significantly affected the risk of osteoporosis. Of these indices, CTDIv (CT) had the highest area under the curve (AUC) (0.735). Correlation analyses of tBMD with cumulative radiation dose revealed weak correlations between tBMD and CTDIv (CT) (*r* ^2^ = 0.194, *P*<0.001).

**Conclusion:**

The prevalence of osteoporosis was significantly higher in post TACE patients than in control subjects. The cumulative radiation dose related to routine dynamic CT studies was a significant contributor to the prevalence of osteoporosis.

## Introduction

The number of computed tomography (CT)-guided interventional procedures has increased because they are less invasive and more cost-effective than open surgery [1]–[3]. Transarterial embolization therapies involve the transcatheter delivery of solid particles into an artery feeding a target tumor for the purpose of blocking its blood supply. These therapies include bland embolization, transarterial chemoembolization (TACE), and chemoembolization using drug-eluting beads. TACE is a method in which chemotherapeutic drugs are combined with embolization particles and then injected into the artery that supplies the tumor. TACE via the hepatic artery has been used as treatment for hepatocellular carcinoma (HCC) in cases where surgical resection is not a viable option or as a means of downstaging HCC to fit within Milan criteria for the possibility of further management with orthotopic liver transplantation. Trials performed by Hong Kong [4] and Barcelona [5] researchers showed a significant increase in survival rates of subjects compared to controls. In some institutions, CT arterial portography and CT during hepatic arteriography are performed to confirm the existence or to evaluate the characteristics of liver tumors during TACE [6]. Additionally, two-dimensional or three-dimensional CT images are acquired to generate a road map of the targets and their positions relative to the interventional instruments. This valuable information provides guidance for the operator to locate the target, plan an interventional path, adjust the interventional instruments, and evaluate the efficacy of the procedure. A priori knowledge of the distribution of contrast material in the tumor from performance of CT arteriography through the selected arterial branch ensures that anticancer drugs and embolic agents are infused effectively.

A concern regarding the use of CT during angiography may be the radiation exposure to the patients, because the number of CT scans performed during TACE with the interventional-CT system is high; around ten times per procedure. It is well recognized that therapeutic irradiation can result in bone damage and may increase fracture risk. The main evidence for the effect of irradiation on fracture risk comes from a long-term follow-up study of two European randomized trials (Stockholm I and II) [7], [8] evaluating the effect of short-course irradiation on patients with operable rectal cancer [9]. Pathologically, vascular injury and decrease of osteoblast cells following irradiation have been reported [10]–[14].

**Figure 1:**
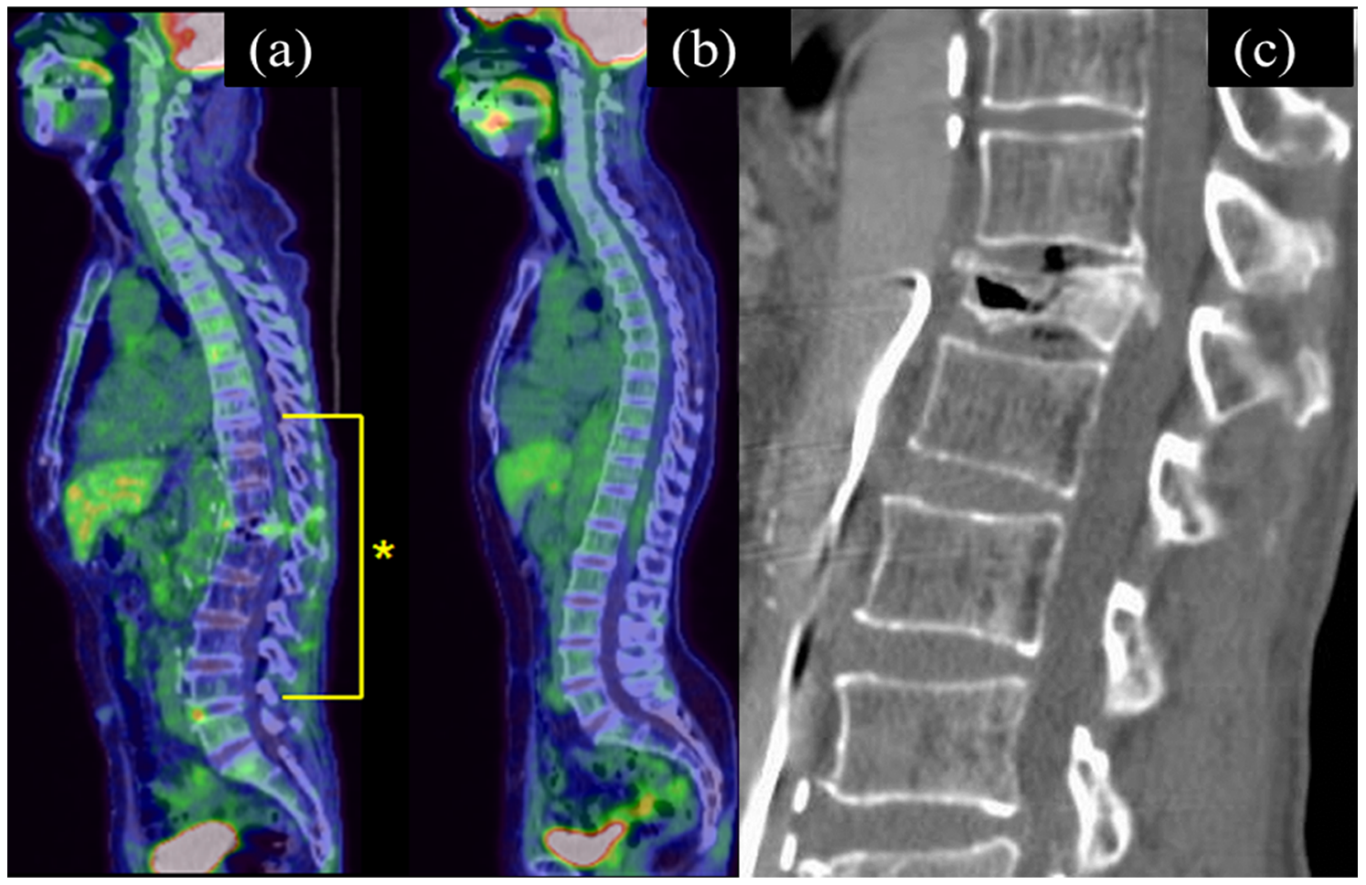
Sagittal reconstructed fluorodeoxyglucose-positron-emission tomography (FDG-PET)-CT image (a) and MDCT image (c) of the spine obtained from a 62-year-old man after eight transarterial chemoembolization procedures for hepatocellular carcinomas. FDG-PET-CT reveals diffusely decreased FDG uptake within the lower thoracic to lumbar spine (*). Sagittal reconstructed FDG-PET-CT image is accompanied by that of a 72-year old woman performed five years after resection of a uterine cervical cancer, in which there is normal spinal FDG uptake, for comparison (b). Sagittal reconstructed CT shows a vertebral fracture of the 12th thoracic vertebra.

Recently, a case of 12th thoracic vertebral fracture that occurred after multiple TACE procedures to treat multiple hepatocellular carcinomas was seen. Fluorodeoxyglucose (FDG)-positron-emission tomography (FDG-PET)-CT performed to screen for pathologic fractures revealed diffusely decreased FDG uptake within the lower thoracic to lumbar spine (Figure 1). This finding gave us the impression that this area might have corresponded to the radiation field during the TACE procedures because the liver and vessels such as hepatic arteries, their branches, superior mesenteric artery, and portal vein, which are repeatedly imaged during TACE procedures, are all located around the same spinal level. Therefore, we hypothesized that radiation exposure from CT scans during angiographies led to decreased bone density and bone strength associated with secondary osteoporosis.

Bone strength and fracture susceptibility are governed in large part by the amount of bone, which can be assessed by dual-energy X-ray absorptiometry measurements of areal bone mineral density. However, many other structural and material properties of bone, including microarchitecture, contribute considerably and independently to fragility [15]–[17]. Recently, high-resolution peripheral quantitative CT [18], [19] and multidetector CT (MDCT) [20], [21] have been used noninvasively to visualize the details of trabecular microarchitecture.

The long-term bone toxicity associated with CT during interventional angiography has received little attention. The purpose of this study was to determine the prevalence of osteoporosis and trabecular microstructural changes using clinical MDCT-based microstructural analysis in patients after TACE with the interventional-CT system to treat hepatocellular carcinoma.

## Materials and Methods

### Ethics Statement

This retrospective, single-institution study was approved by the Institutional Review Board of Hiroshima University Hospital, with a waiver of informed consent. Patient records and information were anonymized and de-identified prior to analyses.

### Subjects

First, consecutive patients who underwent unenhanced abdominal CT performed with a specific CT unit in our institution from September 2010 to January 2014 were selected. Next, a total of 195 of these patients were enrolled in the study after exclusion of subjects with a history of chemotherapy (n = 4), total or partial gastrectomy (n = 4), esophagectomy (n = 1), radiation therapy to the pelvis (n = 1), vertebral fracture (n = 1), chronic steroid use (n = 1), and patients with a gait disturbance caused by cerebrovascular disease (n = 4) or myopathy (n = 1). There were no patients with a history of rheumatoid arthritis, inflammatory bowel disease, chronic obstructive pulmonary disease, or organ transplantation. Patients in the TACE group (n = 81) had HCC with a history of TACE and a diagnosis of either type B or type C hepatitis. Patients in the chronic hepatitis (CH) group (n = 35) had a diagnosis of either type B or type C hepatitis and had no history of any angiographic or CT-guided fluoroscopic procedures. The majority of HCC patients had persistent infection from hepatitis B virus or hepatitis C virus. Therefore, we established the CH group to determine whether there were radiation effects from TACE procedures only. Subjects in the control group (n = 79) were randomly selected from our hospital’s radiology information system; they had undergone MDCT of the body to screen for tumor recurrences other than liver tumors, to rule out internal malignancy, and to diagnose abdominal or back pain. We did not recruit healthy control subjects from the local community for ethical reason.

Tables 1 and 2 summarize the characteristics of these patients.

**Table 1:** Comparison of clinical characteristics and cumulative radiation doses.

Men	TACE	CH	Control
Background data	n = 48	n = 17	n = 33
Age (years)	67.1±9.0	66.3±7.6	66.5±7.1
BMI (kg/m^2^)	23.2±2.8	21.8±2.4	23.8±3.9
Radiation exposure			
CTDIv (CT) (mGy)	350††[50, 1950]	200** [50, 950]	50 [0, 700]
CTDIv (TACE) (mGy)	148 [15, 528]		
DLP (CT) (mGy×cm)	14350†† ^,+^[2000, 78000]	7600 [0, 22900]	4500 [0, 22200]
DLP (TACE) (mGy×cm)	2818 [305, 17985]		
Entrance skin dose (mGy)	790.9 [36, 5911]		
**Women**	**TACE**	**CH**	**Control**
Background data	n = 33	n = 18	n = 46
Age (years)	67.4±7.8	61.7±6.9	62.5±9.3
BMI (kg/m^2^)	23.0±3.4	22.9±4.4	22.5±3.4
Radiation exposure			
CTDIv (CT) (mGy)	374†† ^,^ ** [100, 1650]	250 [0, 1150]	0 [0, 200]
CTDIv (TACE) (mGy)	101 [26, 1723]		
DLP (CT) (mGy×cm)	9450^†,^ ** [2800, 33000]	6900 [0, 36000]	1900 [0, 13000]
DLP (TACE) (mGy×cm)	1880 [489, 6599]		
Entrance skin dose (mGy)	817 [76, 4465]		

Note. Values represent the means ± standard deviation or medians [range].

BMI, body mass index; CTDIv (CT): CTDIv of routine dynamic CT scan; CTDIv (TACE): CTDIv during TACE; DLP (CT): DLP of routine dynamic CT scan; DLP (TACE): DLP during TACE.

**P<0.01, control vs. CH group.

†† *P*<0.01, †*P*<0.05 control vs. TACE group.

+*P*<0.05, TACE group vs. CH group.

**Table 2:** Comparison of bone mineral density of the lumbar spine among the three groups.

Male	TACE	CH	Control
tBMD (mg/cm^3^)	78.6±35.4^†^	82.0±26.5	91.6±24.9
<68 mg/cm^3^ (osteoporosis)	19	6	6
≥68 mg/cm^3^	29	11	27
Prevalence of osteoporosis (%)	39.6	35.3	18.2
**Female**	**TACE**	**CH**	**Control**
tBMD (mg/cm^3^)	60.3±31.0††	76.4±30.6	84.6±29.2
<68 mg/cm^3^ (osteoporosis)	20	9	16
≥68 mg/cm^3^	13	9	30
Prevalence of osteoporosis (%)	60.6	50.0	34.8

†† *P*<0.01, †*P*<0.05, control vs. post TACE.

### TACE Procedure

All patients in the TACE group had adequate hepatic function to undergo TACE. The interventional procedures were performed using an interventional-CT system consisting of a unified CT and angiography unit (Aquilion LB combined with Infinix Celeve-i INFX-8000V, Toshiba Medical Systems, Tokyo, Japan). In addition to angiographic examinations, CT scans were performed during common or proper hepatic arteriography to evaluate hemodynamics in the HCC, map the arterial anatomy, and assess portal flow. Further selective hepatic arteriograms were also performed using CT scanning to confirm the distribution of contrast material in the HCC from the selected arterial branch just before infusing anti-cancer drugs and embolic agents.

The X-ray tube was equipped with a built-in kerma area product (KAP) meter at the collimator exit with capability to display the cumulative KAP for fluoroscopic and radiographic examinations. The KAP is the integral of air kerma (the energy extracted from an X-ray beam per unit mass of air in a small irradiated air volume; for diagnostic X-rays, the dose delivered to that volume of air) across the entire X-ray beam emitted from the X-ray tube. It is a surrogate measure of the amount of energy delivered to the patient [22]. Entrance skin dose was calculated from KAP, beam area, and the calibration coefficient of the KAP meter at the calibrated distance for the focal-to-skin distance. It is important to estimate entrance skin dose during and after fluoroscopically guided interventions because a skin dose that exceeds 15 Gy is thought to be a sentinel event [23]. The volumetric CT dose index during TACE (CTDIv (TACE)) and dose-length product during TACE (DLP (TACE)) were obtained directly from the scanner console. The computed tomography dose index (CTDI) is a measure of the radiation output of the CT slice comprising the quality of the X-rays produced, the type of filtration and the geometry of the X-ray beam including focus size and collimation [24]. CTDIv, which was introduced for modern CT scanners, is obtained from the average CTDI in an irradiated slice divided by a helical pitch. To obtain a dose quantity describing the radiation exposure for a full scan, the DLP was introduced [25], [26]. DLP can be obtained by CTDIv divided by the scan length, which means it depends on the patient’s height.

For each patient, cumulative radiation doses obtained from each index were calculated as the sum of all TACE procedures performed since 2008.

### Imaging by MDCT

All patients were scanned with a 64-section MDCT (LightSpeed VCT; GE Healthcare, Little Chalfont, UK) with spatial resolution equivalent to 16.4 line pair per centimeter at a 2% setting for the modulation transfer function. The collimation was 64×0.625 mm, and table translation speed was 23.4 cm/sec. The tube parameters were 300 mA and 120 kV, with a pitch of 0.586. Unenhanced and three-phase contrast-enhanced helical scans of the entire liver were obtained.

For each patient, the cumulative radiation doses obtained from each of two indices (e.g., CTDIv (CT) and DLP (CT)) were calculated as the sum from all dynamic CT scans since 2008.

### Bone mineral density measurement and MDCT-based microstructural analysis

To obtain tissue bone mineral density (tBMD) data by MDCT, the patients were scanned simultaneously with a bone mineral reference phantom (B-MAS2000; Kyotokagaku Co., Kyoto, Japan) containing calibration objects with equivalent densities of 0, 50, 100, 150, and 200 mg/cm^3^ calcium hydroxyapatite.

Images of the whole lumbar spine obtained by unenhanced scanning were reconstructed using an acquisition matrix of 512×512 and a field of view of 100 mm, resulting in a voxel size of 0.20×0.20×0.16 mm^3^. An edge-enhancing reconstruction kernel (Bone Plus; GE Healthcare, Little Chalfont, UK) was used. The 12th thoracic vertebra (T12) was chosen for the analysis in this study because vertebral fractures typically occur at the thoracolumbar junction (T12 and the first lumbar vertebra) [26], [27].

For microstructural analysis, the volume of interest (VOI) was defined manually as a 10-mm thickness of the central part of the T12 vertebral body to avoid the cortex, the basivertebral foramen, and both endplates.

Microstructural parameters were calculated using a computer program for a three dimensional (3D) image analysis system (TRI/3D-BON; RATOC System Engineering, Tokyo, Japan), as described elsewhere [20]. Briefly, using a volumetric bone mineral density value for trabecular bone, >150 mg/cm^3^ within the bone marrow was extracted. We used a global threshold method. A standardized method of image threshold levels based on the attenuation histogram of a selected region of interest was used to ensure consistency in the image threshold levels across all subjects studied.

The following trabecular microstructural parameters were obtained: apparent trabecular bone volume fraction (app BV/TV), apparent trabecular number (app Tb.N), apparent trabecular separation (app Tb.S), Euler’s number (E), degree of anisotropy (DA), and the structure model index (SMI). Details of these methods are described elsewhere [20], [21] but briefly, bone volume (BV) was calculated using tetrahedrons corresponding to the enclosed volume of the triangulated surface. Total tissue volume (TV) analyzed was the entire marrow area volume including trabecular bone. Apparent BV/TV was calculated from these values. App Tb.S was determined by filling maximal spheres into the structure according to the method described by Hildebrand and Rügsegger [28]. App Tb.N was estimated as a trabecular bone number crossing the line perpendicular to the growing direction of vertebrae based on the plate model [29]. Euler’s number was calculated by using the Euler method of Odgaard and Gundersen [30]. Degree of anisotropy was determined from the ratio between the maximal and minimal radii of the mean intercept length ellipsoid [31]. By displaying the surface of the structure to an infinitesimal amount, SMI was calculated according to the method described by Hildebrand and Rügsegger [28]. The SMI quantifies the plate vs. rod characteristics of trabecular bone. An SMI of 0 reflects a purely plate-shaped bone and an SMI of 3 indicates a purely rod-like bone.

A cutoff value of 68 mg/cm^3^ was determined from a previous study that evaluated tBMD (bone mineral content/tissue volume (BMC/TV)) in 67 patients with osteoporosis using the 64-section MDCT and the same 3D image analysis system [32].

One author (M.T.) performed microstructural analysis, and precision was confirmed as described elsewhere [20].

### Statistical Analysis

The characteristics, cumulative radiation doses, trabecular microstructural parameters, and tBMD of the three groups were compared by the Kruskal-Wallis test followed by the Steel-Dwass test because some indices were non-normally distributed. The Kolmogorov-Smirnov test was used to determine whether values were normally distributed. The prevalence of osteoporosis in the three groups was calculated. For patients in the TACE group, a multivariate general linear model with binomial distribution and logit-link was constructed to identify the predictors of osteoporosis. We calculated the odds ratios for osteoporosis associated with risk factors. To evaluate the diagnostic performance of contributors to osteoporosis, comparisons of the receiver operating characteristic (ROC) curves were performed, and the areas under the curves (AUCs) were calculated. Correlations of tBMD with cumulative radiation dose were determined. The raw datasets of tBMD and CTDIv (CT) showed significant heteroscedasticity and data were transformed to a log scale. Since log-transformed tBMD and CTDIv (CT) were non-normally distributed, the Spearman rank correlation test was used for simple regression analysis. All analyses were performed with a spreadsheet application (Microsoft Office Excel 2010, Redmond, WA, USA) and ROC analysis software (ROCKIT 0.9.1; Charles E. Metz, University of Chicago, Chicago, IL, USA).

## Results

Table 1 summarizes the patients’ characteristics and cumulative radiation doses. In male subjects, CTDIv (CT) and DLP (CT) were significantly greater in the TACE group than in the control group (*P*<0.01). CTDIv (CT) was significantly greater in the CH group than in the control group (P<0.01), and DLP (CT) was significantly greater in the TACE group than in the CH group (*P*<0.05). Patients’ age and BMI were similar among the three groups. In female subjects, CTDIv (CT) and DLP (CT) were significantly greater in the TACE group and the CH group than in the control group (*P*<0.01). Patients’ age and BMI were similar among the three groups.

The prevalence of osteoporosis in the three groups was calculated (Table 2). The prevalence of osteoporosis was significantly greater in the TACE compared to the control group (39.6% vs. 18.2%, P<0.05 for males and 60.6% vs. 34.8%, P<0.01 for females, respectively). The prevalence of osteoporosis in the CH group was less than in the TACE group and greater than in the control group, but these results were not statistically significant.

Results of comparison of microstructural parameters among the three groups are shown in Table 3. Among the microstructural indexes, app Tb.N was significantly lower in the TACE group than in the control group (*P*<0.05). Apparent Tb.S (*P*<0.05 for males, *P*<0.01 for females), structure model index (*P*<0.05 for males, *P*<0.01 for females), and Euler number (*P*<0.01) were significantly higher in the TACE group than in the control group. In female patients, app BV/TV was significantly lower in the TACE group than in the control group (*P*<0.01). These findings indicate that trabecular bones in the patients of the TACE group were fewer, less dense, and more rod-like than in the controls.

**Table 3:** Comparison of microstructural parameters among the three groups.

	Male			Female		
	TACE	CH	Control	TACE	CH	Control
App BV/TV (%)	27.8±13.5	28.6±8.7	31.9±7.8	21.2±10.5**	27.8±10.8	29.8±9.6
App Tb.N (1/mm^3^)	0.34±0.08*	0.36±0.06	0.38±0.05	0.30±0.10*	0.35±0.06	0.36±0.07
App Tb.S (µm)	842±281*	775±147	710±109	1003±392**	811±171	770±195
SMI	1.76±0.53*	1.74±0.39	1.61±0.36	2.00±0.44**	1.75±0.48	1.69±0.40
Euler’s number	−1426±1149**	−1792±1105	−2185±977	−665±879**	−1074±681	−1402±825
Degree of anisotropy	1.41±0.11	1.43±0.12	1.41±0.09	1.48±0.10	1.52±0.20	1.45±0.11

**P<0.01, *P<0.05, control vs. post TACE.

app BV/TV, apparent trabecular bone volume fraction; app Tb.N, apparent trabecular number; app Tb.S, apparent trabecular separation; SMI, structure model index.

Multivariate regression analysis demonstrated that sex, age, and CTDIv (CT) significantly affected the risk of osteoporosis (Table 4). The AUC having the highest discriminatory power to distinguish osteoporotic patients from controls was that for CTDIv (CT) (AUC = 0.735; 95% confidence intervals (CIs), 0.557, 0.732), with an optimal cutoff value of 400 mGy; the sensitivity and specificity were 63.2% (CIs, 0.367, 0.642) and 73.2% (CIs, 0.557, 0.922), respectively. Regarding the same distribution, the AUC for age was 0.692 (CIs, 0.622, 0.771), with an optimal cutoff value of 64 years old; the sensitivity and specificity were 88.6% (CIs, 0.817, 0.972) and 58.8% (CIs, 0.377, 0.811), respectively. Analysis of CTDIv (TACE) revealed no contribution to the risk of osteoporosis.

**Table 4:** Multivariate regression analysis examining the effects of patients’ characteristics and cumulative radiation dose on osteoporosis in the TACE group.

Variable	*β ± Standard error	*P* Value	Odds ratio (CI)
Sex	0.38±0.62	0.001	1.25 (1.65, 7.43)
Age	0.88±0.02	<0.001	1.11 (1.06, 1.17)
CTDIv (CT)	0.85±0.00	<0.001	1.03 (1.00, 1.01)

*β, Standardized partial regression coefficient.

**Figure 2:**
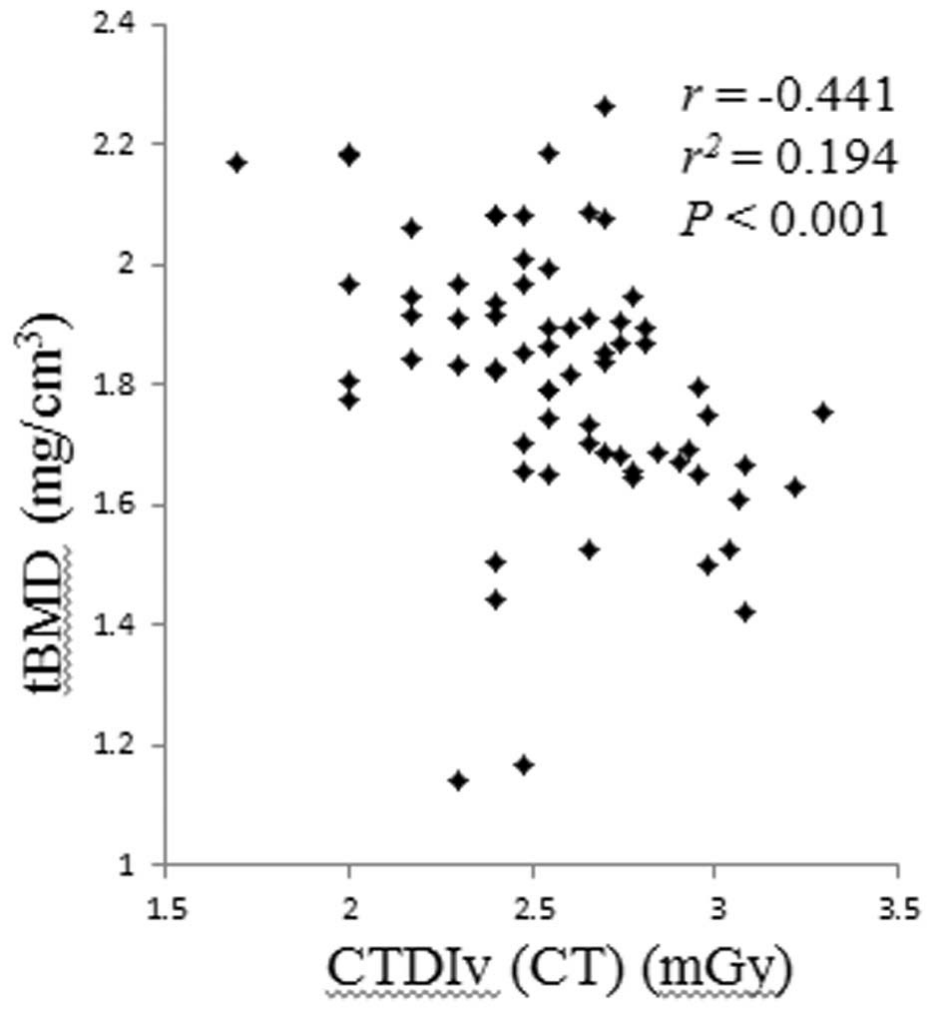
Correlation analysis of tBMD with cumulative radiation dose shows a significant correlation between tBMD and CTDIv (CT).

Correlation analyses of tBMD with cumulative radiation dose showed a weak correlation between tBMD and CTDIv (CT) (*rs* = −0.441, *r* ^2^ = 0.194, *P*<0.001, Figure 2).

**Figure 3:**
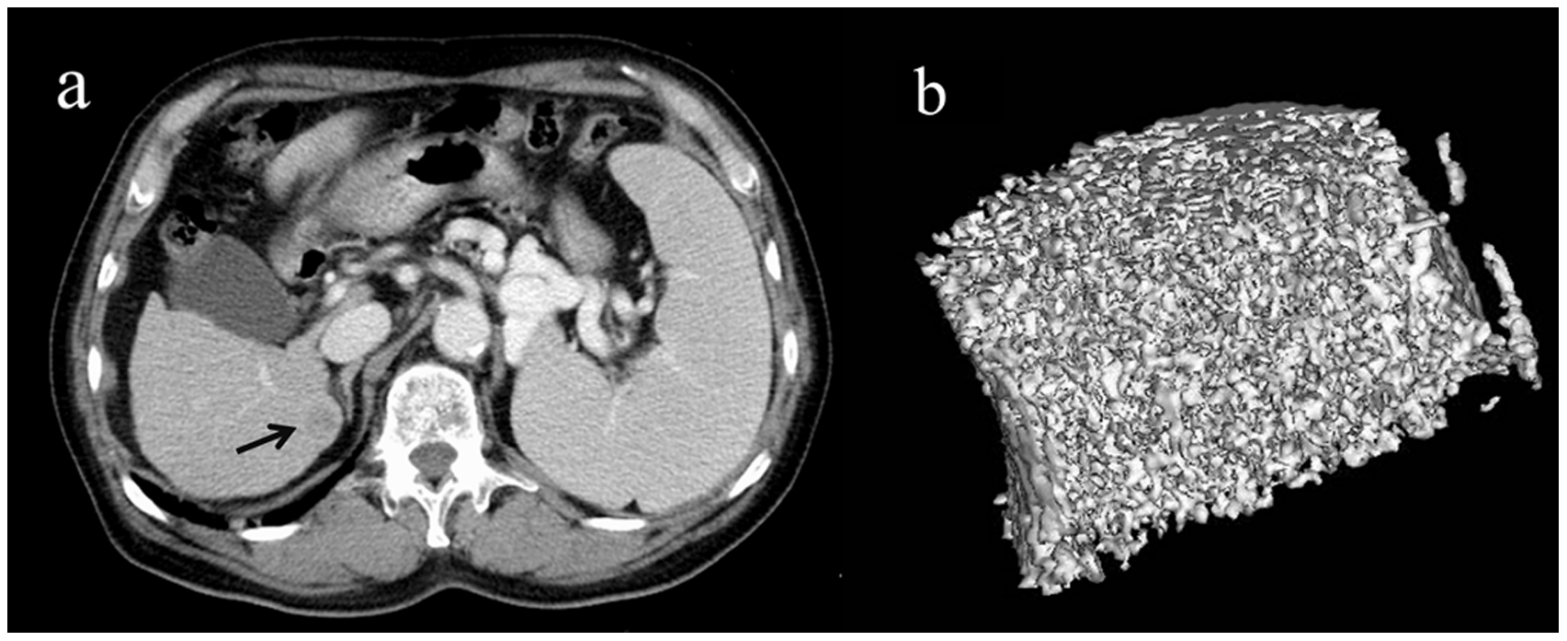
Representative 3D MDCT images of the L3 vertebra obtained from a 71-year-old man after five dynamic CT scans and two transarterial chemoembolization procedures for hepatocellular carcinomas. An axial CT image of the liver shows a recurrence of hepatocellular carcinoma in S7 as a low density area compared to adjacent liver parenchyma(a), (black arrow). The 3D image of the L3 vertebra is shown (b). Tissue bone mineral density (76.8 mg/cm^3^) is normal for age. The image is cut in half along the longitudinal midline.

**Figure 4:**
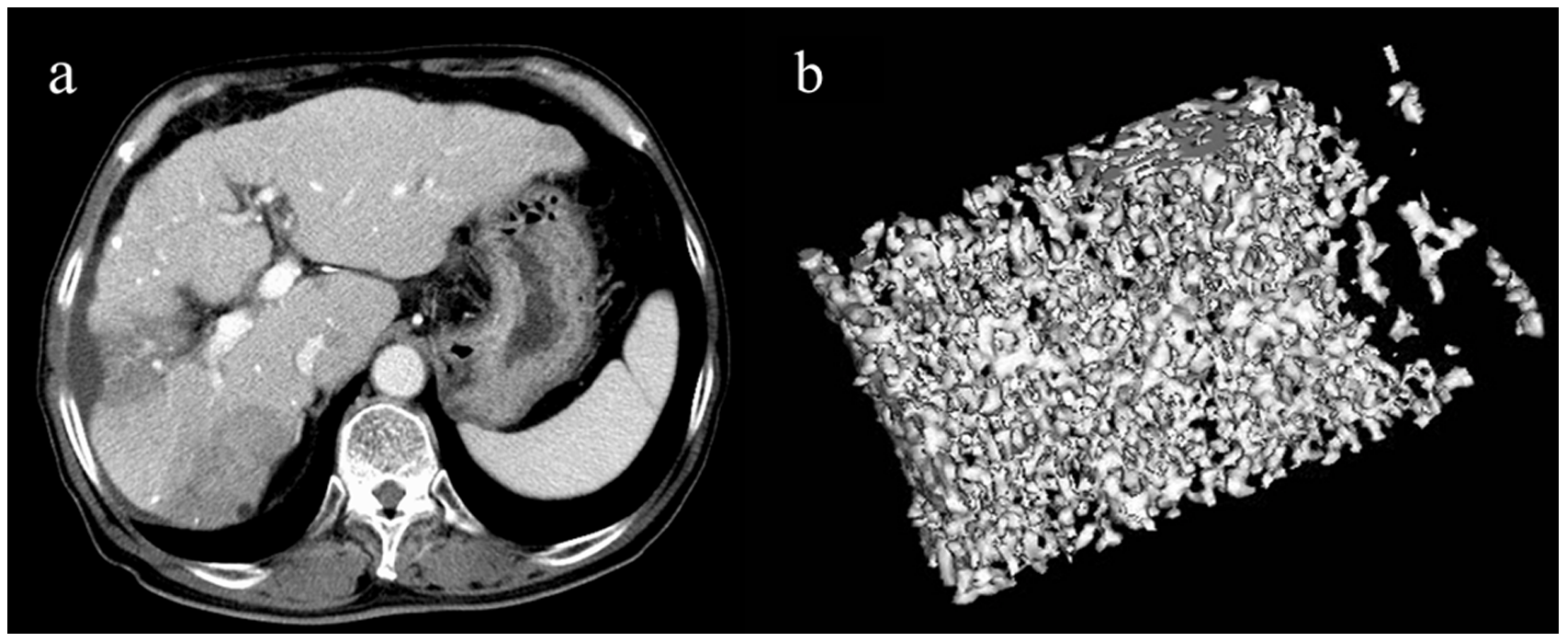
Representative 3D MDCT images of the L3 vertebra obtained from a 71-year-old man after 24 dynamic CT scans and seven transarterial chemoembolization (TACE) procedures for hepatocellular carcinomas. An axial CT image of the liver shows segmental low density areas in the S5 and S7 areas due to previous TACE procedures(a). The 3D image shows sparse trabecular bones (b) compared to those of the patient in Figure 3. Tissue bone mineral density (36.2 mg/cm^3^) is lower than the mean value of control subjects. The image is cut in half along the longitudinal midline.

Representative images are shown in Figures 3 and 4.

## Discussion

In early 2010, the U.S. Food and Drug Administration introduced the “Initiative to Reduce Unnecessary Radiation Exposure from Medical Imaging” [33]. In this report, CT and associated CT-guided interventions were identified as contributors to increasing the radiation dose to patients, and- the authors pointed out that a lack of awareness of radiation-induced injuries to patients among the users of medical radiation may be contributing to an increase of radiation exposure.

In the current study, weak correlations were found between tBMD and the cumulative radiation dose of patients in the TACE group. Bone loss due to high doses of irradiation therapy has been identified in diagnostic radiographic images [34]. However, to the best of our knowledge, no study has demonstrated an association between bone mass and radiation dose from diagnostic imaging procedures such as CT scans.

The primary effect of irradiation on bone is atrophy. Several pathological findings regarding blood vessels were identified in the radiation-induced changes in bone. They included loss of vasculature [10], sub-intimal fibrosis, and hyaline thickening of the media [12], followed by the later replacement of smooth muscle cells [35]. Other investigators have reported a reduction in the number of osteoblast cells following irradiation, which was associated with decreased collagen production and alkaline phosphatase activity [13]. Since both collagen and alkaline phosphatase play a role in mineralization, it has been proposed that this is a pathway to osteopenia [14].

A radiation dose-effect relationship was also demonstrated by several animal experiments. Bone atrophy was detected after irradiation of the femur of rats with doses of 20–25 Gy, and there was also a significant reduction in the relative amounts of calcium and phosphorus in bone, suggesting that atrophy was associated with bone mineral loss [36]. Although the results from animal studies cannot be applied to patients in clinical settings, they could partly explain the relationship between the cumulative radiation dose and tBMD in this study.

On the basis of the multivariate regression analysis, the greatest contributor to the prevalence of osteoporosis among the indices for radiation exposure was the CTDIv (CT) from routine CT examinations to assess or screen for hepatocellular carcinoma. The cumulative radiation dose from angiographic procedures (e.g., the CTDIv (TACE)) did not significantly contribute to the prevalence of osteoporosis. The cumulative radiation dose from routine CT examinations can be up to four times higher than that of angiographic procedures, as shown in Table 1. Thus, a reduction in radiation dose for patients who have to undergo repeat TACE treatments will be best achieved by minimizing the dose from routine CT examinations. The most practical methods to achieve this will be to avoid unnecessary CT scans, to use a lower tube current (mA) or peak beam energy (peak kV), and increase the noise settings for tube current modulation [37], [38].

Among the microstructural indices in this study, app Tb.N and app BV/TV were significantly lower and app Tb.S and SMI were significantly higher in the TACE group compared to the control group.

There have been several reports of results regarding radiation effects on trabecular morphology that are consistent with those of our study. In 2009, Willey et al. [39] investigated the effect on mice of whole-body irradiation of 2 Gy X-rays. They demonstrated that the trabecular microarchitectural properties of L5 included decreased volumetric BMD, BV/TV, and Tb.N and elevated SMI and Tb.S. In 2014, Xu. et al. [40] reported similar results with X-ray radiation of Wistar rats, which had significantly reduced BMD, BV/TV, and Tb.N, but increased SMI.

We did not include trabecular thickness for microstructural analysis in this study. In 2007, correlation analysis of morphological parameters findings of high-resolution peripheral quantitative CT vs. micro-CT was performed by MacNeil et al. [41]. They showed that, among trabecular parameters, trabecular thickness had the lowest correlation coefficient (*r^2^* = 0.59), whereas other high-resolution peripheral quantitative CT-indices, such as trabecular bone volume fraction, trabecular number and trabecular separation, correlated well with the gold-standards methods results (*r^2^*>0.83). In addition, regarding changes in trabecular thickness after radiation exposure, several previous studies have had contradictory findings even using micro-CT. For example, Hamilton et al. [42] reported an insignificant increase in trabecular thickness after radiation exposure of 2-Gy to tibiae and femurs of mice. More recently, Chandra et al. [43] demonstrated a slight but significant increase in trabecular thickness after irradiation with a clinically relevant dose of the proximal tibiae of rats. We used clinical MDCT with spatial resolution of 200 µm×200 µm×160 µm to investigate trabecular bones that are known to have about the same size as the imaging spatial resolution. In the previous study [20] performed by using the same CT scanner and 3D image analysis system, the apparent trabecular thickness was around 700 µm, when many studies using micro-CT report an average Tb.Th of 150–200 µm. We speculate that the actual Tb.Th is overestimated by clinical MDCT due to the combined effects of limited spatial resolution and missing thin trabeculae during the thresholding step. Thus, we think that an evaluation of Tb.Th using clinical CT is inappropriate.

There were several limitations to this study. First, there are several other factors that could cause osteoporosis in the CH- and TACE groups. Risk factors for osteoporosis include advanced age, low body mass index, underlying disease (rheumatoid arthritis, inflammatory bowel disease, chronic obstructive pulmonary disease, history of organ transplantation), smoking, excessive alcohol consumption, and high glucocorticoid dose [44]. Glucocorticoid-induced osteoporosis is the most common and severe form of iatrogenic osteoporosis [44]–[47]. In patients with glucocorticoid-induced osteoporosis, the loss of bone mineral density occurs at the rate of approximately 3% yearly after the first year [44]. In an animal study [48], peripheral quantitative CT analysis revealed glucocorticoid-induced BMD loss of approximately 7% in wild-type mice. On the other hand, in a rat study [43], radiation from micro-CT scans following the irradiation generated in a clinically relevant range led to more reductions in BMD (26% in BMD and 37% in BV/TV). In fact, we excluded patients with a history of chronic steroid use; in addition, patients’ characteristics including age and BMI were similar among the three groups in this study. Thus, we attribute the reduced tBMD in the present study mainly to radiation exposure. Second, there might be some error in the results of this study due to the low spatial resolution of clinical MDCT, although the results of several previous reports regarding radiation effects on trabecular morphology are consistent with those of our study. Validation of microstructural parameters obtained using clinical MDCT by comparing with results using the gold-standard method, micro-CT, is needed during future studies. Third, the radiation dose per each single CT scan or TACE was not identical, and the intervals between these procedures varied across individuals in this study. One clinical study showed the effects of changes in the dose per fraction on the radiation response of mature bone of post mastectomy patients [49]. Spontaneous rib fracture was significantly higher in the group treated with a larger dose/fraction than in the more standard lower dose/fraction group. The patients who underwent repeat TACE in this study tended to have shorter between-scan intervals than control subjects. Such interindividual variability could have affected our results.

In conclusion, the prevalence of osteoporosis detected with MDCT was significantly higher in post TACE patients than in control subjects. The cumulative radiation dose related to routine dynamic CT studies was a significant contributor to the prevalence of osteoporosis. There is a weak relationship between local irradiation to bone and reduced bone density. Based on our study, reduction of the radiation dose for HCC patients might be achieved by minimizing the dose from routine CT examinations; unnecessary CT scans should be avoided.
